# Microarray profiling emphasizes transcriptomic differences between hippocampal *in vivo* tissue and *in vitro* cultures

**DOI:** 10.1093/braincomms/fcab152

**Published:** 2021-07-08

**Authors:** Declan King, Paul A Skehel, Owen Dando, Katie Emelianova, Rona Barron, Thomas M Wishart

**Affiliations:** 1 Centre for Discovery Brain Sciences, UK Dementia Research Institute, The University of Edinburgh, Edinburgh EH8 9JZ, UK; 2 Centre for Discovery Brain Sciences, UK Dementia Research Institute, The University of Edinburgh, Edinburgh EH8 9XD, UK; 3 School of Health Sciences, Queen Margaret University, Edinburgh EH21 6UU, UK; 4 College of Medicine and Veterinary Medicine, The Roslin Institute and Royal (Dick) School of Veterinary Studies, University of Edinburgh, Easter Bush, Midlothian EH25 9RG, UK

**Keywords:** hippocampal cultures, transcriptome, microarray, neuronal

## Abstract

Primary hippocampal cell cultures are routinely used as an experimentally accessible model platform for the hippocampus and brain tissue in general. Containing multiple cell types including neurons, astrocytes and microglia in a state that can be readily analysed optically, biochemically and electrophysiologically, such cultures have been used in many *in vitro* studies. To what extent the *in vivo* environment is recapitulated in primary cultures is an on-going question. Here, we compare the transcriptomic profiles of primary hippocampal cell cultures and intact hippocampal tissue. In addition, by comparing profiles from wild type and the PrP 101LL transgenic model of prion disease, we also demonstrate that gene conservation is predominantly conserved across genetically altered lines.

## Teaser

Currently, 17 862 publications (PubMed) reference cell cultures; however, the biological relevance of cell cultures to their *in vivo* tissue counterparts is still open to question. King et al. conducted a direct transcriptional comparison of cultures and intact tissue. The authors showed transcriptomic fingerprints in tissue and culture were not comparable.

## Graphical Abstract

**Graphical Abstract:**
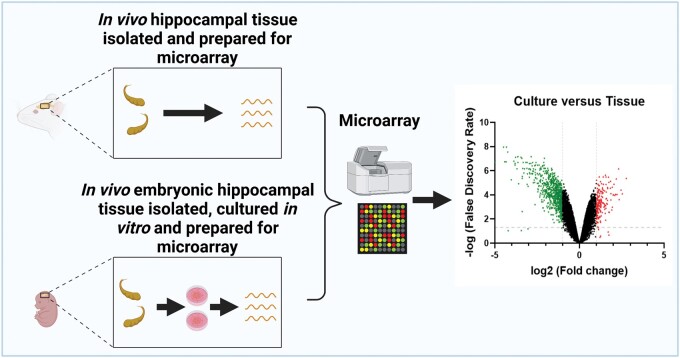

## Introduction

The complex interconnected structure of the mammalian brain and its anatomical location protected by the skull presents particular challenges for the study of cellular and molecular processes. *In vitro* cell cultures attempt to recapitulate the basic cellular environment of the brain in a more experimentally amenable context. Primary hippocampal cell cultures are regularly used as a supplement model to depict the brain's composition in a readily accessible and manipulatable arrangement. Indeed, PubMed currently cites 17 862 publications (1973–2021) associated with hippocampal cultures, and often strong conclusive inferences are drawn from these studies. Although these approaches are considered suitable model platforms, the biological relevance of cultured hippocampal cells to their *in vivo* counterparts is still open to question. One investigation of transcription in dorsal root ganglia and superior cervical ganglia during neurite outgrowth and regeneration, described commonalities in gene expression transcriptomics, relating to regenerating neurons between *in vitro* and *in vivo* models.^1^ Similar gene expression profiles have also been detected in developing hippocampus *in vivo* and primary hippocampal neurons undergoing differentiation both *in vivo* and *in vitro*.^2–4^ Conversely, genome-wide expression analysis of cell lines has indicated dramatic differences in comparison to relevant tissues of origin.^5^ Remaining studies are inconclusive and describe both similarities and differences of biological processes between neural cells grown *in vitro* and *in vivo*.^6^ These conflicting results suggest further studies are required to establish the full utility of cultured hippocampal cells as an *in vitro* model platform. Here, we compare transcriptomic profiles in acutely dissected hippocampal tissue and primary hippocampal cell cultures from both wild type (WT) animals (129/Ola) and a transgenic model of neurodegeneration based on the PrP 101LL mutation.

The 101LL model was included in this study as currently a majority of culture-based studies are being carried out to address questions about the nature of neuronal stability following specific genetic alterations/mutations and/or neurodegenerative challenge. We sought to confirm if the degree of transcriptomic similarity holds true in murine models genetically altered with a single amino acid mutation. One such mouse line was available in our laboratory namely the PrP mutant (101LL, 129/Ola background)^7^ containing a single point mutation of the *Prnp* gene (proline to leucine, modelling Gerstmann–Sträussler–Scheinker disease). This 101LL mutation is not pathological but is known to show altered susceptibility to disease associated protein misfolding.^8^ Therefore, in this current study, we also sought to investigate gene expression changes in the 101LL model relative to WT. Findings suggest that RNA isolated from acutely dissected hippocampal tissue and mature *in vitro* primary cultures provided transcriptomic molecular fingerprints that were not comparable. This was the case for both WT and 101LL genotypes. Direct comparison between genotype (WT and mutant 101LL) revealed no (tissue) or minimal (cell) significant transcriptomic changes indicating transcript profiles were conserved across WT and 101LL genotypes.

These findings broaden our understandings of the biological relevance of cultured hippocampal neurons to their *in vivo* tissue counterparts and transcript changes identified here could be used to drive real progress for future therapeutic investigations using *in vitro* cultures.

## Materials and methods

### Mouse lines

All experiments were conducted under Home Office project licence (2010–2015 PPL 60-4125: 2015–2017 PPL 70-8523) within the regulations of the Animals (Scientific Procedures) Act 1986. Study numbers A820 and A821 were approved by Roslin’s Animal Welfare and Ethical Review Body. WT (129/Ola) mice were obtained from Jackson laboratories. 101LL knock in transgenic mice (129/Ola background, single point mutation, proline to leucine at codon 101 in *Prnp* gene) were generated in-house using a double replacement gene targeting strategy.^7^^,^^9^ WT mice were homozygous for the WT *Prnp* gene (101PP) and 101LL were homozygous for the P101L mutation.

### Production of primary neuronal cell cultures

Primary hippocampal neuronal cultures were prepared based on previously described methods^10–16^ with minor modifications indicated below. Day 17 embryos (E17) were used according to previous protocols.^11^^,^^17^ As this was a time sensitive protocol, embryos were pooled from whole litters irrespective to sex. Once dissected hippocampi were transferred into 222 μl Trypsin (2.5% 10×, Life Technologies) and 20 μl Deoxyribonuclease I (5 mg/ml, Sigma) and incubated at 37°C for 20 min. The tissue was then washed twice in 10 ml pre-heated growth media [500 ml basal medium eagle (Gibco) containing 50 ml heat inactivated Horse Serum (Gibco), 8 ml 32.5% Glucose solution (Sigma), 5 ml Sodium Pyruvate 100 mM (Gibco), 5 ml N2 Supplement (Gibco) and 5 ml Penicillin–Streptomycin (10 000 U/ml, Gibco)]. Samples were then disrupted by trituration in growth media and plated on poly-l-lysine coated six-well plastic dish at a density of 400 000 cells per well (poly-l-lysine, Sigma–Aldrich) plates. Plates were incubated at 37°C/5% CO_2_ for 4 h. Growth media was removed and replaced with an equal volume of serum free media [500 ml neurobasal media (Gibco), 10 ml B27 supplement 50× (Gibco), 5 ml l-Glutamine (200 mM, Gibco), 5 ml Penicillin–Streptomycin (10 000 U/ml, Gibco)]. One-third of media was replaced with fresh pre-warmed serum free media every 3 days and cells were cultured to 8 days *in vitro* (DIV8).

### Immunostaining of primary cultures

Cell media was removed from six-well plates and cells were incubated with 4% paraformaldehyde (v/v) for 15 min at room temperature. This was followed by three 5-min washes with Dulbecco’s phosphate-buffered saline containing Ca^2+^ and Mg^2+^ (Gibco).

About 1–2 ml of ice-cold methanol was added to the cells for 10 min with incubation at −20°C followed by a 5-min incubation with 0.3% Triton-X (Sigma, v/v) at room temperature. Again, wells were washed three times in Dulbecco's phosphate-buffered saline at 5-min intervals then blocked for 1 h at room temperature using Fc Block (CD16/32, BioLegend). Primary antibodies were incubated overnight in 5% Goat serum (Gibco, v/v) at 4°C (concentrations listed in Table 1). Cells were washed three times in Dulbecco's phosphate-buffered saline (Gibco), and secondary antibodies diluted in 5% goat serum (v/v, Table 1) were added for 1 h at room temperature in complete darkness followed by a further three washes as above. Pre-labelled poly-l-lysine six-well plates (Biocoat Cell Environments) were imaged using a LSM710 inverted confocal microscope (Zeiss).

**Table 1:** Primary and secondary antibodies used for immunolabelling experiments

Primary antibody	Marker/concentration	Secondary/concentration	Supplier
Anti-MAP2 (Ab5392)	Dendritic, 20 mg/ml, 1/2000	Goat Anti-Chicken IgY (Alexa Fluor 488) 1/500	Both Abcam
Anti-GFAP (Ab53554)	Astrocyte, 0.5 mg/ml, 1/500	Donkey Anti-Goat IgG (Alexa Fluor 555) 1/500	Both Abcam
Anti-Iba1 (019–19741)	Microglia, 1mgml, 1/1000	Goat Anti-Rabbit IgG (Alexa Fluor 594) 1/500	Wako Abcam
Synapsin 1 (Ab64581)	Pre-synaptic, 1 mg/ml, 1/200	Goat Anti-Rabbit IgG (Alexa Fluor 594) 1/200	Both Abcam
PSD-95 (Ab99009)	Post-synaptic, 1 mg/ml, 1/200	Goat Anti-Mouse IgG (Alexa Fluor 647) 1/200	Both Abcam

### Acutely dissected hippocampal tissues

Hippocampal tissues were obtained from brains of mice at postnatal Days 6–7. A non-Schedule 1 termination of each individual postnatal pup involved decapitation followed by immediate brain removal and immersion into RNAlater RNA stabilization Reagent (Qiagen). Tissues were isolated each time from three pup brains (six hippocampi) from the same litter of pups which were then combined to produce one individual sample. As this was a time sensitive protocol, brains were pooled irrespective to gender. This was replicated four times per genotype.

### Cell lysis and RNA extraction

Ribonucleic acid (RNA) was extracted from both cell culture (Day 8, DIV8) and Day 6 mouse hippocampal tissue samples using the RNeasy Plus Micro Kit (Qiagen) according to manufacturer’s instructions. The rationale here was that E17 (assuming gestation period of 20–21 days) harvested embryos would be cultured *in vitro* for 8 days to provide a more comparable developmental stage to that of day 6 tissue *in vivo* cells. For cell cultures, RNA extractions were always pooled in cases where more than one well was cultured from the same batch of embryos and this was counted as one sample, which was replicated four times per genotype (WT and 101LL). In total, 16 samples were generated (4 WT cell, 4 101LL cell, 4 WT tissue and 4 101LL tissue) and RNA integrity number values of 9 or above were obtained for each sample (Agilent TapeStation System) indicating high quality intact RNA was isolated.

### Microarray hybridization and labelling

RNA labelling and hybridization were carried out by Edinburgh Genomics, University of Edinburgh (https://genomics.ed.ac.uk/ Accessed 13 July 2021). For microarray, cDNA was produced using the Ambion WT expression kit (Invitrogen) and accordingly labelled using the GeneChip WT terminal labelling kit (Affymetrix).

Approximately 3 µg of fragmented, biotin-labelled cDNA was hybridised to a Mouse Gene 2.1 ST array plate (Affymetrix) using the Gene Titan instrument (Affymetrix) and standard Affymetrix protocols.

### Data QC and normalization

Affymetrix microarray processing produced 16 (4 WT cell, 4 101LL cell, 4 WT tissue and 4 101LL tissue) probe cell intensity data files which can be downloaded from https://doi.org/10.7488/ds/3016 Accessed 13 July 2021. Robust Multichip Average pre-processing was performed on these raw microarray intensity datasets for background subtraction, quantile normalization and summarization, using the R package ‘oligo’ (R package version 1.52.1).^18^ A principal component analysis (PCA) plot was generated by PCA of log transformed, normalized expression data, and a clustered heatmap by calculating the Manhattan distance between sample pairs. Transcript clusters with very low expression, with no gene annotation, or with ambiguous gene annotation were subsequently removed. Differential expression was then performed using the R package ‘limma’ (R package version 3.44.3)^19^ (Supplementary material Files 1–4; 1_diff_expr_WT_cell_vs_tissue, 2_diff_expr_101LL_cell_vs_tissue, 3_diff_expr_cell_WT_ vs_101LL, 4_diff_expr_tissue_WT_ vs_101LL) and gene ontology (GO) analysis was performed using the R package “topGO” (R package version 2.40.0)^20^ (Supplementary material Files 5 and 6; 5_go_all_bp_WT_cell_vs_tissue, 6_go_all_bp_101LL_cell_vs_tissue, https://doi.org/10.7488/ds/3015 Accessed 13 July 2021). Standard filtering parameters included false discovery rate (FDR) *P*-value <0.05. Ingenuity Pathway Analysis (IPA, Qiagen) was used to search through gene lists and determine genes involved in well documented canonical signal transduction pathways.^21^

### Real-time quantitative reverse transcription PCR

cDNA samples (16 samples in total, 4 WT culture, 4 101LL culture, 4 WT tissue and 4 101LL tissue) at a concentration of 25 ng/μl were generated. Mastermix for real-time quantitative reverse transcription PCR (RT-qPCR) using Primerdesign was as follows; 1 µl resuspended primer mix (300 nM in a 20 μl reaction); 10 µl 2X PrecisionPLUS mastermix; 4 µl RNAse/DNAse free water. All reactions were carried out using the Stratagene Mx3005p system and SYBR green mastermix (Primerdesign/Agilent technologies). Reactions were done using 96-well PCR plates (ABgene) and optical caps (Applied Biosystems). Each sample was loaded in triplicate. To identify suitable reference/housekeeping genes, the GeNorm PCR kit (Primerdesign) was used as described in manufacture’s protocol. Two cDNA samples from each representative group (WT, 101LL cultures; WT, 101LL tissue) were analysed to identify the most suitable candidate reference gene over all samples for use in normalization experiments.

Results from the GeNorm PCR kit were analysed using the Biogazelle qbase^+^ analysis software. Analysis results showed average expression stability of 12 reference targets ranking according to expression stability. Tyrosine 3-monooxygenas/tryptophan 5-monooxygenase (*Ywhaz*) was stably expressed across all 16 microarray samples and therefore was selected as reference/housekeeping gene for all RT-qPCR runs.

### Validation experiments

Primers were selected based on target genes of interest and included Laminin alpha 1 (*Lama1*), Midline 1 (*Mid1*), Transforming growth factor beta induced (*Tgfbi*), Myocyte Enhancer Factor 2 C (*Mef2c*) and Transthyretin (*Ttr*). Relative changes in gene expression were calculated using the Delta Delta Ct (ΔΔC_T_) method.^22^^,^^23^

### IMARIS software analysis of immunolabelled hippocampal culture images

IMARIS software (Bitplane) allowed for data visualization and analysis of confocal microscopy datasets, in the format ‘czi’. For each image or channel within an image the intensities of all voxels based on fluorescent signal were analysed using default standard IMARIS formulas that calculated mean, standard deviation and sum intensities (intensities do not have any units) and therefore these values were used for relative comparison of targets of interest across all comparative images.

### Statistical analysis

All graphs and statistics were generated in GraphPad Prism 9. Normality and Lognormality (Anderson–Darling, D’Agostino–Pearson and Shapiro–Wilk) tests were performed prior to any statistical testing. If data sets passed the normality test, a one-way ANOVA with Tukey’s/Sidaks post-hoc was carried out. When data sets did not pass the normality test a non-parametric Mann–Whitney test was carried out. For statistical tests, *P *<* *0.05 was used for significance. All ANOVA tests were presented with *F* and *P* values for main effects.

Significant effects between groups, identified by post-hoc analysis, were displayed visually on graphs and recorded in text as *P*-values. All data were plotted as means with 95% CI for normal distribution and medians with 95% CI for non-parametric data.

### Data availability statement

Affymetrix microarray probe cell intensity data files and all differential expression comparison files including GO analysis have been deposited in The University of Edinburgh, College of Medicine & Veterinary Medicine, Royal (Dick) School of Veterinary Studies, The Roslin Institute, Functional Genetics and Development, DataShare and are available at: https://doi.org/10.7488/ds/3016 Accessed 13 July 2021 and https://doi.org/10.7488/ds/3015 Accessed 13 July 2021.

### Compliance with ethical standards

All applicable international, national and institutional guidelines for the care and use of animals were followed. All procedures performed in studies involving animals were in accordance with the ethical standards of the institution at which the studies were conducted. The article does not contain any studies with human participants performed by any of the authors.

## Results

### Characterization of neuronal cell cultures confirm suitability of model for *in vivo* comparison

**Figure 1:**
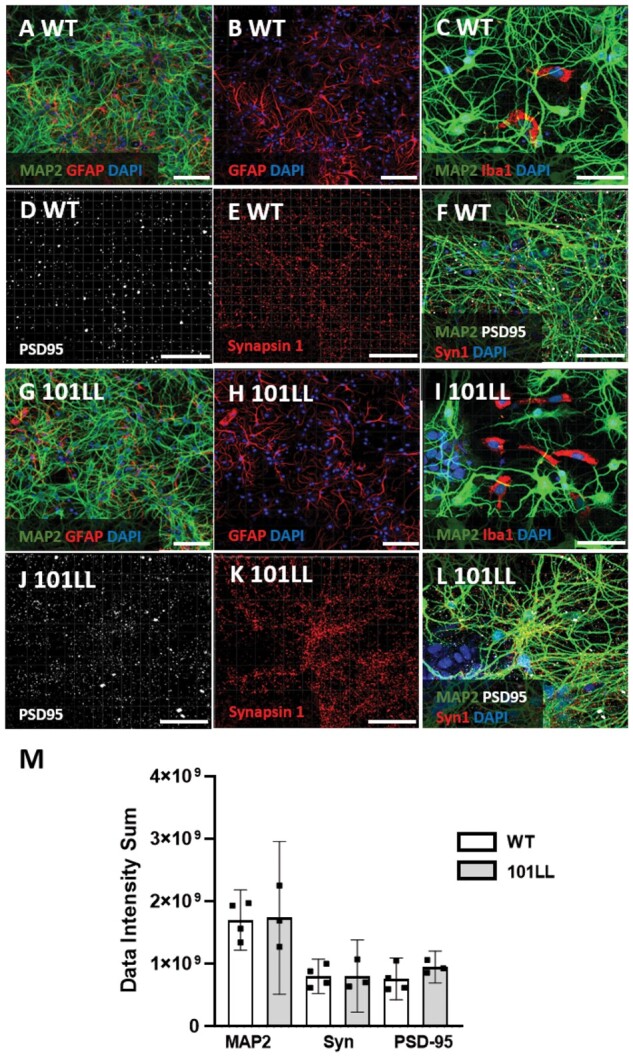
**Characterization of hippocampal cultures confirmed neuronal maturity.** Primary cultures consisted of mature neuronal networks supported by glial cells. (**A–F**) WT cultures. (**A**) Neuronal populations were present as indicated using microtubule associated protein 2 (MAP2/Green; DAPI/Blue) and were supported by glial cells namely astrocytes confirmed by Glial Fibrillary Acidic Protein immunolabelling (GFAP/Red; DAPI/Blue) (**B**) and (**C**) microglial cells confirmed by Ionized calcium-binding adapter molecule 1 (Iba1/Red; DAPI/Blue). Primary cultures developed into mature neuronal synaptic networks confirmed by (**D**) post-synaptic marker, Anti-Postsynaptic Density protein 95 (PSD-95/White) and (**E**) Pre-synaptic marker, Synapsin 1 (Syn1/Red). (**F**) Merged image of synaptic markers and MAP2. (**G–L**) 101LL cultures immunolabelled in same order as WT. (**M**) Fluorescent signal data intensity sums generated from immunolabelled MAP2, Synapsin1 and PSD-95 using IMARIS based on days *in vitro* 8 (DIV8) were plotted from both genotypes. (**A–L**) Representative WT/101LL cell culture images, DIV8, 30 000 cells plated on PDL plastic plates, Scale bar 50 μm (**A–B**; G-H) 30 μm (**C–F; I–L**), Zeiss LSM 710. (**M**) Fluorescent signal data intensity sums, one-way ANOVA was conducted to compare intensity means of immunolabelled DIV8 cultures WT and 101LL. There were significant differences between antibody means as expected *F*(5,15) = 10.29, *P* = 0.0002. Sidaks multiple comparison test between groups showed no significant differences. MAP2 mean difference (md) −3.66E + 07; 95% CI of differnece (95% CI) −5.94E + 08 to 5.21E + 08; Adjusted *P*-value (Adj PV) 0.997; Synapsin1 md −5.50 + 06; 95% CI −5.62E + 08 to 5.51E + 08; Adj PV >0.999; PSD-95 md −1.88 + 08; 95% CI −7.46E + 08 to 3.68E + 08; Adj PV 0.758. Graph plotted mean with 95% CI, *n* = 4 WT; *n* = 3 101LL.

Cultures developed highly branched neuronal networks by DIV8 (days *in vitro*, standard timeline is 8 days), evident from microtubule associated protein 2 immunolabelling and were supported by glia (Fig. 1A–C; G–I). Neuronal maturity was confirmed by the presence of both pre (synapsin1) and post-synaptic (PSD-95) protein markers (Fig. 1D–F; J–L). To investigate if WT and 101LL cultures were comparable regarding cellular profile, immunolabelled images of MAP2/Synapsin1/PSD-95 DIV8 cell cultures were processed using IMARIS software. Data intensity comparisons established from fluorescent signal for each target protein showed no significant differences between WT and 101LL cell cultures indicating both had similar cellular profiles (Fig. 1M). This also reinforced reproducibility of the culture method used across genotype. Overall, these cultures were similar to those reported in the literature^17^^,^^24^^,^^25^ and were appropriate for use in the comparative molecular fingerprinting experiments proposed here.

### Microarray profiling establishes transcriptomic differences between hippocampal *in vivo* tissue and *in vitro* cell cultures comparisons

**Figure 2:**
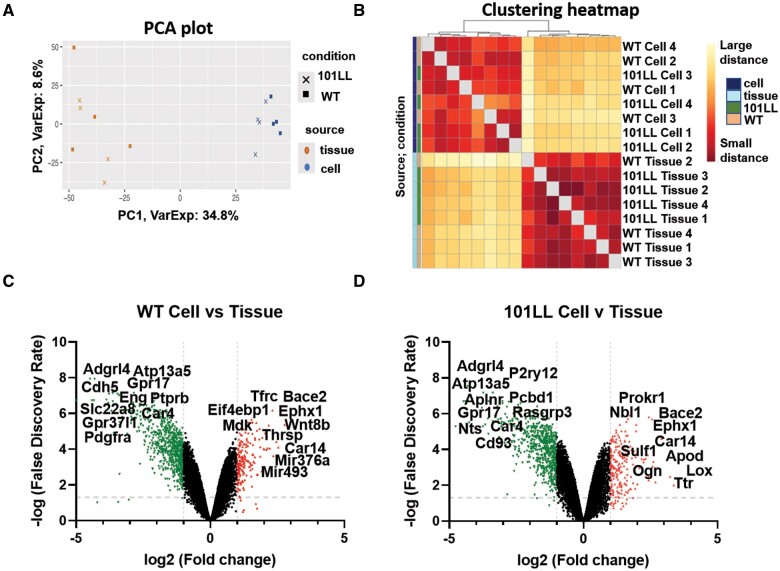
**Microarray analysis highlighting transcriptomic profile differences exist between hippocampal *in vivo* tissue and *in vitro* cell cultures.** (**A**) Principal component analysis (PCA) plot of log-transformed expression data demonstrates clear separation in sample distribution between *in vivo* and *in vitro* platforms suggesting both are not comparable on a transcriptomic level. Both WT and 101LL genotypes correlated in a similar fashion irrespective of context. (**B**) Clustering heatmap for all 16 calibrated samples clearly demonstrated a difference in gene expression patterns existed between cell (upper, dark blue) and tissue samples (lower, light blue). (**C**) Scatter plot of WT cell versus tissue was constructed to ascertain the magnitude of gene changes between platforms. All genes (represented by individual dots/triangles) above the dotted horizontal line passed the standard filtering parameters (FDR *P*-value 0.05, −log 1.3) and therefore were significant. In this case, 5199 differentially expressed genes (DEGs) were identified of which 2706 were down regulated and 2493 were upregulated. Applying more restrictive filtering to include a fold change (FC) of two represented here by the dotted vertical lines (−1, 1 log2), 830 DEGs were identified (682 down/green; 148 up/red). (**D**) 101LL cell versus tissue produced a similar scatter profile where 4677 (2527 down, 2150 up) were identified at FDR *P*-value 0.05. When including FC2 this number was reduced to 856 DEGs (660 down/green and 196 up/red). For both graphs, top 20 gene names (based on *P*-value/FC) are displayed and highlight similar gene changes were occurring across genotypes.

The microarray platform was employed here to compare gene expression in both *in vitro* hippocampal cell culture and *in vivo* hippocampal tissue RNA extracts to establish to what extent these contexts were or were not comparable. A PCA plot (first and second components) generated from microarray analysis of all 16 *in vivo* and *in vitro* samples (4 WT cell, 4 101LL cell, 4 WT tissue and 4 101LL tissue) was used to visualize patterns associated with these datasets (Fig. 2A).

The visualization plot generated confirmed a clear pattern and separation was evident between cell (orange) and tissue (blue) groups of arrays analysed by principal component. This was independent of genotype.

These observations were further supported by clustering all 16 samples on a heatmap (Fig. 2B), where again differences in gene expression patterns were evident between cell and tissue platforms. To ascertain the magnitude of gene changes between *in vivo* and *in vitro*, standard filtering of datasets was carried out using an FDR *P*-value of <0.05. To visualize these changes, scatter graphs were produced accordingly. Scatter plots of both WT cell versus tissue and 101LL cell versus tissue (Fig. 2C and D) clearly show numerous differentially expressed genes (DEGs) were detected using standard filtering parameters (FDR *P*-value <0.05). Here each dot/triangle resembles a single gene, genes above the dotted horizonal line were significantly changed. For WT cell versus tissue 5199 DEGs (2706 down and 2493 up) were detected (Fig. 2C). When applying additional fold change parameters of two (donated by dotted vertical lines) 830 DEGs were detected of which 682 were downregulated and 148 were upregulated. In comparison, 101LL cell versus tissue produced similar results with 4677 DEGs identified (2527 down and 2150 up) at FDR *P*-value <0.05 (Fig. 2D). When a fold change of two was included, this number was decreased to 856 DEGs (660 down and 196 up). As highlighted on both scatter graphs similar genes were changing across both genotypes, including downregulation of *Adgrl4*, *Atp13a5, Car4* and upregulation of *Bace2, Car14, Ephx1.*

Comparison of 101LL versus WT tissue identified no DEGs using *P*-value <0.05 indicating the single point mutation in the *Prnp* gene did not change baseline transcript profile *in vivo*. Whilst comparing 101LL versus WT cell, 5 DEGs were identified, and all were upregulated in the 101LL genotype. One of these genes namely Midline 1 (*Mid1*), is associated with microtubule stabilization^26^ suggesting that neurons may be less stable in the 101LL genotype thus, increasing expression of *Mid1* may be protective. Indeed, it is known that these transgenic animals show altered susceptibility to disease and this may well explanation observations here.^8^ Collectively, it is evident here that baseline transcriptomic profiles are analogous between WT murine models and genetically altered models with a single amino acid mutation.

### IPA highlights multiple affected pathways

To investigate biological functions associated with DEGs identified and the pathways they influence, significantly filtered datasets from both WT and 101LL cell versus tissue were analysed using topGO and IPA. Biological GO terms identified using topGO were numerous (Supplementary material File 5 and 6, https://doi.org/10.7488/ds/3015 Accessed 13 July 2021) and were comparable across WT cell versus tissue and 101LL cell versus tissue datasets.

**Figure 3:**
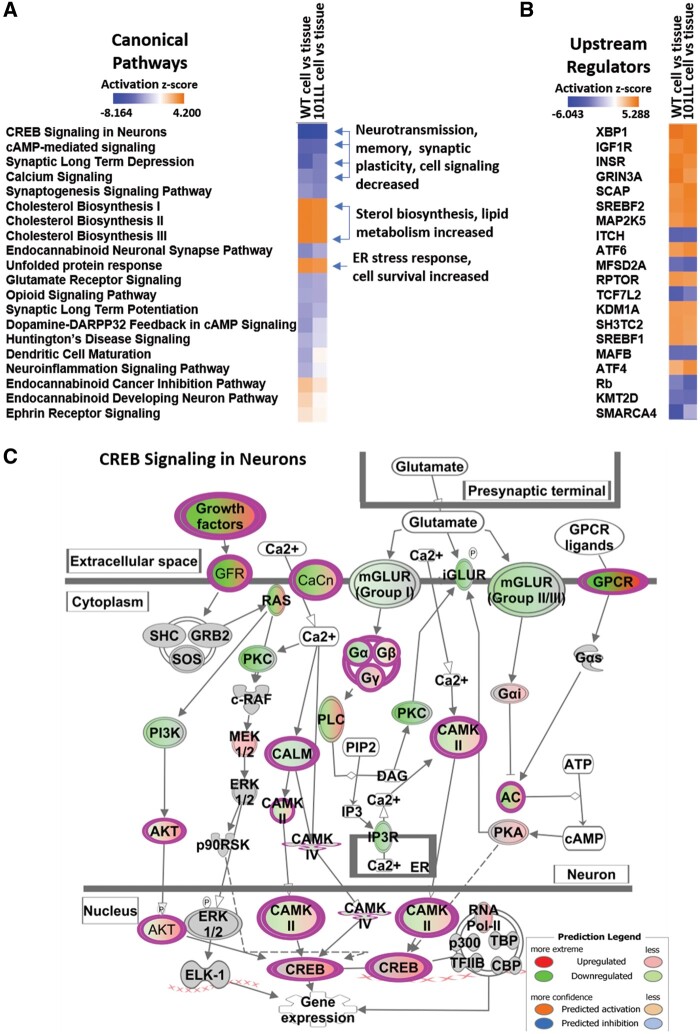
**IPA identified numerous canonical pathway changes *in vitro*.** (**A**) Using standard filtering parameters (*P*-value <0.05), across both WT cell versus tissue and 101LL cell versus tissue datasets, IPA generated a list of canonical pathways of which the top significant 20 are listed here (filtered specifically for brain related pathways). Boxes in blue donate pathways that were inhibited and boxes in orange donate activated pathways. (**B**) Top 20 upstream regulators display gene consistency across comparisons. (**C**) CREB signalling in neurons was identified as the most significantly changed canonical pathway in these dataset comparisons and here we show the specific genes involved in this CREB signalling cascade.

Briefly, top biological processes identified *in vitro* and similar to both datasets included the following, adenylate cyclase-activating G protein-coupled receptor signalling pathway, MAPK cascade, learning or memory, long-term synaptic potentiation and calcium ion transport which were all downregulated. Biological processes upregulated *in vitro* included cholesterol biosynthetic process, regulation of cell growth, forebrain neuron development, regulation of Wnt signalling pathway and endoplasmic reticulum unfolded protein response. IPA also provided an alternative means of data analysis and interpretation. This software is built on a very comprehensive and manually curated knowledge database and therefore provides unique capabilities to identify the most significant pathways, whether activated or inhibited from our experimental data. Evidence from Fig. 3A (FDR *P*-value <0.05) shows top canonical pathways identified in IPA are conserved between genotypes and are predominantly inhibited/downregulated *in vitro*. Canonical pathways identified here complement topGO results where neurotransmission, cell signalling, and memory were all inhibited *in vitro*. Activated pathways are associated with cholesterol synthesis Wnt signalling and ER unfolded protein response. To gain further insights into gene expression changes in our datasets we included a heatmap of the top 20 upstream regulators (Fig. 3B). Here, we show genes identified were consistently expressed across both WT cell versus tissue and 101LL cell versus tissue.

The most significant canonical pathway identified was CREB signalling in neurons and this was shown to be inhibited *in vitro* (Fig. 3A). The CREB as a nuclear transcription factor binds to CRE (cAMP response element, which is also shown the be inhibited *in vitro*), and regulates transcription activity of its downstream substrates. This in turn regulates neuronal processes, including metabolism and survival. Several gene changes were identified in this cascade and as shown in Fig. 3C were all predominantly downregulated.

### Validation experiments

**Figure 4:**
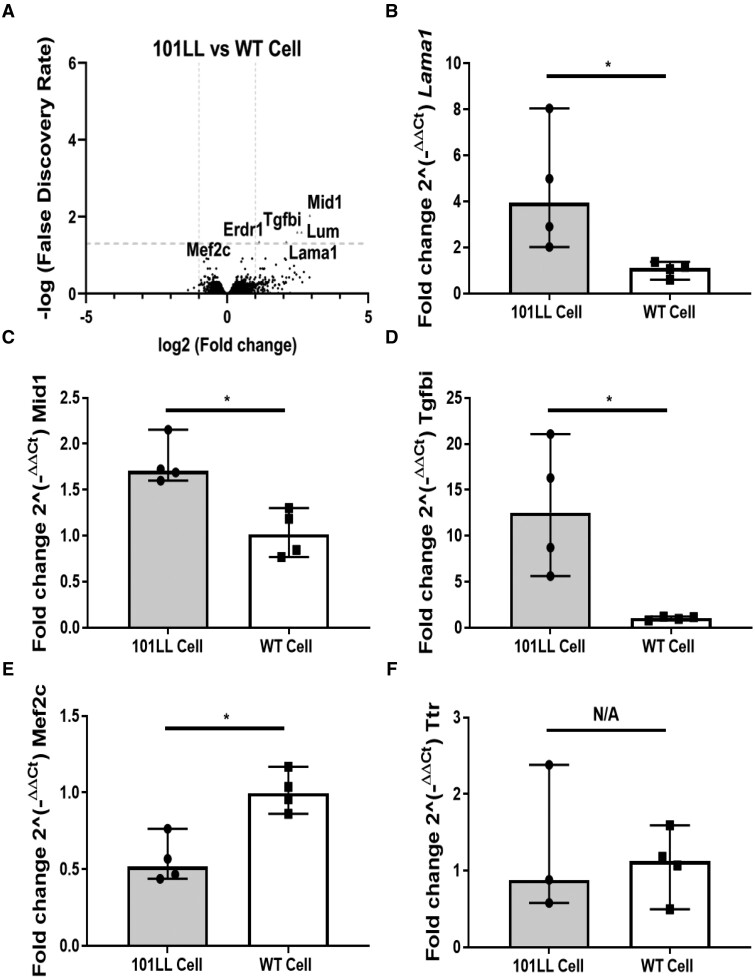
**Validation of microarray expression datasets.** (**A**) Comparing 101LL versus WT cell 5 DEGs (FDR *P*-value <0.05) were identified by microarray analysis (all upregulated in 101LL genotype). Gene names displayed on scatter graph for clarity (above the horizontal dotted line donates significant genes FDR *P*-value <0.05, and above vertical dotted line fold change of two). (**B–D**) RT-qPCR validation of selected genes namely *Lama1, Mid1* and *Tgfbi*. Profiles shown here match microarray data profiling. (**E–F**) *Mef2c* a downregulated gene and *Ttr* an unchanged gene was also included in validation for robustness. Both genes again validated microarray data. RT-qPCR reference gene *Ywhaz*, fold changes from RT-qPCR were calculated using 2-ΔΔCt method. Nonparametric Mann–Whitney *t*-tests were carried out in all cases, graphs plotted as median with 95% CI, *n* = 4, *n* = individual samples. Note for *Ttr* 101LL cell *n* = 3.

Microarray expression results (Table 2) were validated using RT-qPCR based on significant DEGs identified *(Lama1, Mid1* and *Tgfbi*) in the 101LL versus WT cell cohort (Fig. 4A). For completeness and robustness additional genes that were either downregulated (*Mef2c*) or not changed between comparisons (*Ttr*) were also included in this validation. As evident from Fig. 4B–F similar expression trends between all comparisons were found using RT-qPCR thus validating microarray data, and confirming results presented here were accurate.

**Table 2:** Gene fold changes identified from microarray analysis matched RT-qPCR expression profiles exactly

Gene	LogFC 101LL v WT cell	Adjusted *P*-value	RT-qPCR Mann–Whitney
*Lama1*	2.10	0.04	0.03
*Mid1*	2.93	0.01	0.03
*Tgfbi*	2.48	0.03	0.03
*Mef2c*	−0.67	0.13	0.03
*Ttr*	0.98	0.93	0.99

## Discussion

This study has attempted to address the lack of data detailing the molecular composition of cell culture platforms used to model and infer upon their intact *in vivo* neuronal counterparts. Studies by others have attempted similar investigations, however, direct comparisons between identical genotypes from both *in vivo* and *in vitro* contexts was never carried out, instead separate studies were combined to study transcriptomic changes between both. For example, one study focussed on murine gene expression in developing hippocampus *in vivo*,^2^ and a second study on expression profiling of primary hippocampal neurons undergoing differentiation *in vitro*.^3^^,^^4^ Both studies were then combined for subsequent comparisons. Results showed *in vitro* and *in vivo* expression profiles were similar. These findings contradict the results presented here however their study was comparing primary cultures obtained from CD1 outbred mice which have more genetic diversity^4^ with hippocampal tissue from C57BL/6 inbred mice which are almost genetically identical.^2^ Therefore, these studies did not represent an accurate direct comparison of *in vitro* and *in vivo* platforms. Another study using a similar approach (combining two separate studies) described both similarities and differences of biological processes between neural cells grown *in vitro* and *in vivo*.^6^ However, these results were based on rat neural cells obtained from commercial sources (*in vitro*) compared to mouse acutely purified neural cells (*in vivo*), implying these data involved studying cross-species transcriptomic comparisons. Thus, although *in vitro* and *in vivo* comparisons have been carried out previously, the studies were restrictive.

Studies presented in both the introduction and here in the discussion suggest complex *in vivo* tissues consisting of multiple cell types have similar gene expression profiles to neuronal cultures. These findings are questionable as differences in *in vitro* cultures would be expected due to the simplicity of the platform where many of these transcriptional changes would be driven by the emission of cell types present *in vivo*. In this study, a direct comparison between *in vivo*/*in vitro* models and genotype was done and as expected changes in gene expression between organized *in vivo* tissues were detected. However, for a more accurate transcriptional profile comparison, single-cell RNA-seq comparing gene expression of individual cell types such as neurons between both *in vivo* and *in vitro* platforms could be carried out and this was a limitation of our study here which only used bulk RNA-seq.

Gene expression changes presented here indicated that primary hippocampal cultures and acutely dissected hippocampal tissues were not comparable and numerous biological pathways were perturbed *in vitro*. Pathways relating to memory again would be expected to be inhibited in cultures as shown here however many other pathways relating to a broad range of biological pathways were also disturbed including neurotransmission, cell signalling, cholesterol synthesis, Wnt signalling and ER unfolded protein response. Again, studies utilizing *in vitro* platforms to study such pathways should be cautious in their interpretations of results.

Interestingly, gene expression was not altered between WT and mutant genotype (apart from 5 DEGs in cell) indicating a single amino acid mutation may not alter detectable transcriptomic changes between transgenic and WT models. Thus, by comparing profiles from WT and the PrP 101LL transgenic model of prion disease, we demonstrate that gene conservation is predominantly conserved across genetically altered lines. It is possible by using other technologies further transcriptional changes could be detected as microarray hybridization is restricted to predefined transcripts/genes and this is one limitation of the study presented here. RNA-sequencing, for example, could profile the entire transcriptome of these models and therefore could be used to detect more subtle transcriptional changes.

In conclusion, we have shown adequate evidence that primary hippocampal cultures are significantly different to their *in vivo* tissue counterparts at a transcriptional level, and one should be cautious when planning and interpreting data from primary cultures. This study also provides a unique transcriptome resource and a list of canonical pathways that are significantly altered *in vitro*. These insights should help in future experimental planning or to re-access previously published data based on neuronal cell culture models.

## Supplementary material

Supplementary material is available at *Brain Communications* online.

## Abbreviations

DIV =: days *in vitro*
DEGs =: differentially expressed genes
FDR =: false discovery rate
GO =: gene ontology
IPA =: ingenuity pathway analysis
PCA =: principal component analysis
RT-qPCR =: real-time quantitative reverse transcription PCR
WT =: wild-type
